# White Tea Aqueous Extract: A Potential Anti-Aging Agent Against High-Fat Diet-Induced Senescence in *Drosophila melanogaster*

**DOI:** 10.3390/foods13244034

**Published:** 2024-12-13

**Authors:** Yan Huang, Miaoyuan He, Jianming Zhang, Shilong Cheng, Xi Cheng, Haoran Chen, Guangheng Wu, Fang Wang, Shaoxiao Zeng

**Affiliations:** 1 College of Tea and Food Science, Wuyi University, Wuyishan 354300, China; yanhuangfst@wuyiu.edu.cn (Y.H.); hmy202203@163.com (M.H.); zjm0308@163.com (J.Z.); cslong163@163.com (S.C.); wyxychengxi@wuyiu.edu.cn (X.C.); 2 College of Food Science, Fujian Agriculture and Forestry University, Fuzhou 350002, China; chenhaoran6054@163.com; 3 Fujian Provincial Key Laboratory of Quality Science and Processing Technology in Special Starch, Fujian Agriculture and Forestry University, Fuzhou 350002, China; 4 Fujian Provincial Key Laboratory of Eco-Industrial Green Technology, College of Ecology and Resources Engineering, Wuyi University, Wuyishan 354300, China; wguangheng@163.com; 5 College of Food Science and Technology, Ningde Normal University, Ningde 352100, China; fangcaopianpian@163.com

**Keywords:** white tea aqueous extract, anti-aging, *Drosophila melanogaster*, high-fat diet, RNA-seq

## Abstract

White tea has been scientifically proven to exhibit positive biological effects in combating chronic diseases, including cancer, metabolic syndrome, etc. Nevertheless, the anti-aging activity and mechanism of white tea on organisms exposed to a high-fat diet remain unexplored. Herein, we prepared a white tea aqueous extract (WTAE) from white peony in Fuding and assessed its in vivo antioxidant and anti-aging effects by employing a *Drosophila melanogaster* senescence model induced by lard, delving into the underlying molecular mechanisms through which the WTAE contributes to lifespan improvement. Notably, the WTAE significantly extended the lifespan of *Drosophila* fed a high-fat diet and partially restored the climbing ability of *Drosophila* on a high-fat diet, accompanied by increased activities of copper-zinc superoxide dismutase, manganese-superoxide dismutase, and catalase and decreased lipid hydroperoxide levels in *Drosophila*. Furthermore, transcriptomic analysis indicated that the WTAE countered aging triggered by a high-fat diet via activating oxidative phosphorylation, neuroactive ligand–receptor interactions, and more pathways, as well as inhibiting circadian rhythm-fly, protein processing in the endoplasmic reticulum, and more pathways. Our findings suggest that WTAE exhibits excellent inhibitory activity against high-fat diet-induced senescence and holds promising potential as an anti-aging agent that can be further developed.

## 1. Introduction

Aging is marked by a gradual deterioration in tissue and organ function, contributing to various age-related disorders such as cardiovascular diseases, neurodegenerative disorders, and cancer, which exert a significant influence on global human health [1]. The mechanisms underlying aging are multifactorial. The free radical aging theory, initially put forward by Harman [2], is widely acknowledged. Mitochondria generate reactive oxygen species (ROS), including superoxide anion radicals (∙O_2_^−^) and hydroxyl radicals (∙OH), etc., which play essential roles in normal biological activities [3]. Under normal physiological conditions, the organism’s antioxidant enzyme defense system, comprising superoxide dismutase (SOD), catalase (CAT), and glutathione peroxidase (GSH-Px), is capable of eliminating these free radicals and keeping them in a dynamic equilibrium [4]. However, exposure to external adverse stimuli leads to an accumulation of free radicals, resulting in mitochondrial damage and dysfunction, ultimately leading to cellular and tissue deterioration and contributing to aging [5]. The free radical aging theory posits that excessive build-up of free radicals acts as a primary senescence driver. Hence, enhancing antioxidant enzyme activity and maintaining the organism’s free radical balance represent promising anti-aging strategies.

The prevalence of obesity has surged globally, driven by dramatic changes in lifestyle and unrestricted consumption of high-fat diets [6]. Chronic adherence to high-fat diets is associated with adverse health effects, including accelerated aging and reduced lifespan [7]. Consequently, it is imperative to explore effective strategies to mitigate the adverse consequences of high-fat diets. Various studies have endeavored to identify interventions that counteract or attenuate aging and the shortened lifespan induced by high-fat diets. Notably, investigations have revealed the lifespan-extending properties of several plant compounds and natural plant extracts in diverse model organisms exposed to high-fat diets, and these substances are regarded as excellent natural antioxidants [8,9]. Among the six principal tea types in China, white tea stands out as a noteworthy contender. Characterized by a straightforward production process involving the plucking of new buds and young leaves, followed by withering and drying, white tea preserves an elevated level of bioactive substances [10]. In vitro experiments have convincingly evidenced the capacity of white tea extract to effectively restrain adipogenesis and stimulate lipolysis activity in human subcutaneous (pre)-adipocytes [11]. Furthermore, experiments utilizing a dietary obesity mouse model have suggested that white tea aqueous extract may confer anti-obesity effects by enhancing energy expenditure, promoting fatty acid oxidation, and inhibiting fatty acid synthesis [9]. However, despite these promising findings, the potential anti-aging activity of white tea aqueous extract on organisms subjected to high-fat diets and the underlying mechanisms remain unclear.

The fruit fly (*Drosophila melanogaster* Meigen) is an outstanding model for probing the impacts of diet on metabolism, behavior, aging, and longevity [12]. Previous and recent investigations have consistently demonstrated that high-fat diets exert a significant increase in mortality and expedite the aging process in *Drosophila*. These diets also adversely affect climbing behavior and in vivo antioxidant activity [13]. In light of these findings, our study aims to explore the potential lifespan-promoting effects of WTAE when administered to *Drosophila* subjected to a high-fat diet. Specifically, we prepared a white tea aqueous extract from Baimudan in Fuding and employed *Drosophila melanogaster* offered a high-fat diet as an aging model to assess the in vivo antioxidant and anti-aging properties of the WTAE from the aspects of lifespan, locomotor ability, and in vivo antioxidant activity. Additionally, the anti-aging mechanisms of WTAE were revealed through RNA-seq and qRT-PCR analyses for *Drosophila*. The findings indicate the potential application of WTAE as an antioxidant and anti-aging agent within the fields of food and healthcare.

## 2. Materials and Methods

### 2.1. Chemicals and Materials

In this investigation, the white tea variety utilized was Bai Mudan produced in 2023 and purchased from Chueco Tea Co., Ltd. in Wuyishan (Wuyishan, China). Standard chemicals, including gallic acid (GA), catechin (C), (−)-epicatechin (EC), (−)-Epigallocatechin (EGC), (−)-Epicatechin-3-gallate (ECG), (−)-epigallocatechin-3-gallate (EGCG), (−)-catechin gallate (CG), (−)-catechin gallate (GCG), and caffeine (CAF) were obtained from Shanghai Yuanye Bio-Technology Co., Ltd. (Shanghai, China). Acetonitrile and acetic acid of chromatography grade were purchased from Sigma-Aldrich (Shanghai) Trading Co., Ltd. (Shanghai, China). Yeast powder was acquired from Beijing BioDee Biotechnology Co., Ltd. (Beijing, China). The kits for estimating protein content, SOD1, SOD2, CAT, and lipid peroxide (LPO) were obtained from Nanjing Jiancheng Biochemical Co., Ltd. (Nanjing, China). Acid red was purchased from Hefei BASF Biotechnology Co., Ltd. (Hefei, China).

### 2.2. Preparation of the WTAE

A total of 500 g of high-quality white tea was blended with 7500 mL of boiled distilled water in a stainless steel pot. The mixture was subjected to micro-boiling extraction for 30 min. Subsequently, the mixture was filtrated using a double-layer silk cloth to obtain the tea soup, which was then concentrated using rotary evaporation at 60 °C to one-tenth of its original volume. Next, the concentrated tea soup was subjected to vacuum freeze-drying for 48 h, resulting in the production of the white tea aqueous extract (WTAE), which was stored at −20 °C after vacuum heat sealing.

### 2.3. Chemical Analysis of the WTAE

The contents of total polyphenols, total flavonoids, free amino acids, soluble proteins, and soluble sugars in the WTAE were determined according to the Folin-phenol colorimetric method, aluminum trichloride colorimetric method, ninhydrin colorimetric method, Coomassie brilliant blue colorimetry method, and anthrone sulfuric acid colorimetry method, respectively. The principal catechin compounds, encompassing C, EC, EGC, ECG, EGCG, and CG, as well as GCG, GA, and CAF, were quantified using a high-performance liquid chromatography (HPLC) system (Shimadzu LC-20A, Shimadzu Cooperation, Tokyo, Japan) equipped with a C18 column (5 μm-Agilent TC-C18, 250 mm × 4.6 mm). Measurements were performed at a wavelength of 278 nm, with a flow rate of 1 mL/min and a 10 μL injection volume at 35 °C. Mobile phase A consisted of acetonitrile, acetic acid, edetate disodium, and water (45:10:1:444), while mobile phase B comprised acetonitrile, acetic acid, edetate disodium, and water (400:10:1:89). The elution gradient was as follows: 100% A was maintained for 10 min, followed by a transformation to 68% A and 32% B within 15 min. Subsequently, 68% A and 32% B were sustained for 10 min before returning to 100% A.

### 2.4. Culture of Drosophila melanogaster

The fly strain utilized in this research was the wild-type *Drosophila melanogaster w*^1118^, and it was acquired from the Third Institute of Oceanography, Ministry of Natural Resources (Xiamen, Fujian, China). All flies were kept under conditions of 25 °C, 50% relative humidity (RH), and a 12 h dark/light cycle. The standard diet, as previously reported [14], was slightly modified and comprised 34.0 g of cornmeal, 26.0 g of sucrose, 3.0 g of agar, 3.0 g of yeast, and 300 mL of distilled water. Additionally, 1.6 mL of propionic acid was included to hinder mold growth. For the high-fat diet, 10% lard (*w*/*v*) and 1% Tween-20 (*v*/*v*) were incorporated into the basal diet and thoroughly mixed. This study exclusively selected male flies whose hormone effects were smaller in comparison to those of female flies, as hormone levels could regulate the aging process [15,16].

### 2.5. Lifespan Assay

Newly eclosed male flies (8–10 h old) were randomly assigned into five groups, each consisting of 200 flies, and raised in 10 vials with 20 flies per vial. The non-lard control group (NCTL) was maintained on the basal diet. The lard control group (LCTL) was provided the basal diet enriched with 10% lard. The three experimental groups, LWTAE1, LWTAE5, and LWTAE10, were based on the lard control diet supplemented with 1 mg/mL, 5 mg/mL, and 10 mg/mL of the WTAE, respectively. The number of deceased flies was recorded every three days, and the surviving flies were moved to fresh vials containing the corresponding diet until all perished.

### 2.6. Feeding Assay

Dietary intake was assessed using the method previously reported by Wang et al. [16]. Briefly, 240 newly emerged male flies were gathered and raised on the basal diet for six days, followed by 2 h of starvation on filter paper soaked with distilled water. Subsequently, males were placed on either the NCTL diet (*n* = 60, 3 vials), the LCTL diet (*n* = 60, 3 vials), or the LWTAE5 diet (*n* = 60, 3 vials), with 0.2% sulforhodamine B sodium salt (Acid red) incorporated into the diets for an additional 2 h. Food intake was quantified by assessing the degree of abdomen redness, graded on a scale ranging from 0 (colorless abdomen) to 5 (completely red abdomen).

### 2.7. Fly Weight Measurement

Changes in body weight serve as an essential indicator for evaluating food intake. The body weight of male flies fed three diets, NCTL, LCTL, and LWTAE5, was recorded on days 0, 10, and 35, respectively. Specifically, on the designated days, 400 fruit flies (*n* = 100 per replicate, with 4 replicates per group) from each treatment were gently anesthetized with light ether and weighed. The average body weight of male flies within each group was then calculated.

### 2.8. Climbing Ability Assay

Fruit flies exhibit a natural inclination to climb upwards in enclosed spaces, making it feasible to assess their motor function by measuring climbing ability, quantified as the number of flies scaling a fixed distance within a given time [13]. In brief, 20 male flies were introduced into a glass vial and timed for 20 s to assess their climbing behavior. At the end of each trial, the number of males capable of reaching a vertical distance of 8 cm or higher was recorded. The climbing assay was conducted three times on days 10, 25, and 35 for males reared on one of the three diets: NCTL, LCTL, and LWTAE5.

### 2.9. In Vivo Antioxidant Activity

Male flies were reared on the NCTL, LCTL, or LWTAE5 diet for 0, 10, and 35 days, after which they were weighed following light ether anesthesia and then frozen at −80 °C. Subsequently, 100 mg of the male mixture was homogenized in a 9-fold cold saline buffer, followed by centrifugation at 10,000 rpm for 10 min at 4 °C. According to the kit instructions, the total protein content in *Drosophila* was assessed, and the activities of SOD1, SOD2, and CAT, alongside the levels of LPO, were determined by diluting the supernatant to achieve the required concentration.

### 2.10. RNA-Seq

RNA-seq analyses were conducted by Personal Biotechnology Co., Ltd. situated in Shanghai, China. Total RNA was extracted from 30-day-old male *Drosophila melanogaster* individuals subjected to normal, LCTL, and LWTAE5 diets, and RNA amount and purity were quantified using NanoDrop NC-2000 (Thermo Scientific, Waltham, MA, USA). PolyA-structured mRNA was enriched from total RNA, and the mRNA was broken into fragments of about 300 bp in length. Utilizing these fragments as templates, double-stranded cDNA was synthesized to construct the library. Employing second-generation sequencing technology on the Illumina sequencing platform, these libraries underwent paired-end sequencing.

### 2.11. Analysis of Differential Genes and Functional Enrichment

DESeq software (1.39.0) was used to perform differential expression of genes (DEGs) analysis. Genes with a *p*-value of <0.05 and a fold-change value of ≥2 were considered significantly differentially expressed between the two groups. Statistical enrichment analyses for Gene Ontology (GO) and Kyoto Encyclopedia of Genes and Genomes (KEGG) pathways of DEGs were executed via the topGO and KAA programs, respectively.

### 2.12. Quantitative Real-Time PCR

To further validate our transcriptome sequencing findings, a total of 14 specific candidate genes were selected for quantitative real-time PCR (qRT-PCR) analysis. Total RNA was extracted from entire flies utilizing TRIzol reagent (Takara, Kusatsu, Shiga, Japan) in accordance with the manufacturer’s protocol. Subsequently, cDNA synthesis was conducted using a Reverse Transcription Kit (Takara, Kusatsu, Shiga, Japan), and qRT-PCR was performed with SYBR Green (Takara, Kusatsu, Shiga, Japan). Ribosomal protein 49 (*Rp49*) served as the designated housekeeping gene for mRNA expression calculations, as detailed in Table S1. The qRT-PCR data analyses were processed using a GFX 96 ConnectTM Optics Module (Bio-Rad Laboratories, Hercules, CA, USA). The relative quantification of the target gene was ascertained through the ΔΔCT method. 

### 2.13. Statistical Analysis

Statistical analysis of the experimental data was executed using GraphPad Prism 8.0 (GraphPad Software, San Diego, CA, USA). Values are presented as mean ± standard deviation (SD). Lifespan data underwent survival analysis through the Kaplan–Meier test [log-rank (Mantel–Cox) test] and are presented as survival curves. A student’s *t*-test was adopted for comparing two groups. For the comparison of multiple groups, we applied a one-way analysis of variance (ANOVA) followed by Tukey’s Honestly Significant Difference (HSD) test. *p*-values < 0.05 were regarded as significant.

## 3. Results and Discussion

### 3.1. Chemical Compositions of the WTAE

**Figure 1:**
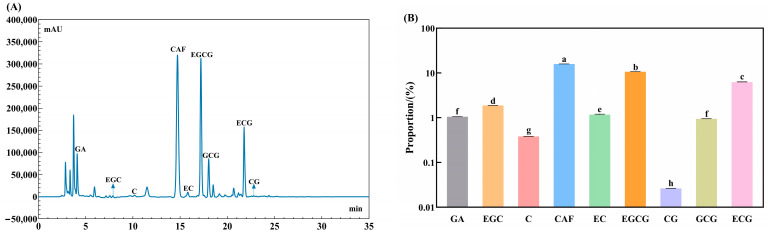
HPLC spectrum (**A**) and percentage (**B**) of catechins and caffeine in the white tea aqueous extract (WTAE). Data are expressed as mean ± SD. Significant differences at *p <* 0.05 among various ingredients are denoted by different lowercase letters (Tukey’s test).

The contents of total polyphenols, total flavonoids, free amino acids, soluble proteins, and soluble sugars are shown in Table 1. The WTAE showed higher levels of total polyphenols (22.92%) and soluble proteins (17.50%). The primary polyphenol compounds and caffeine in the WTAE were identified by HPLC, with the quantitative analysis presented in Figure 1: (1) GA (1.05%); (2) EGC (1.87%); (3) C (0.38%); (4) EC (1.17%); (5) EGCG (10.62%); (6) CG (0.03%); (7) GCG (0.94%); (8) ECG (6.22%); and (9) CAF (15.8%). Notably, the WTAE was rich in CAF and EGCG, followed by ECG. The anti-obesity effects of EGCG and CAF have been widely acknowledged [17,18], and the experimental findings have shown that the former can restrain fat accumulation in *Drosophila melanogaster* of the *Canton-S* wild-type [19] and extend the lifespan of obese rats by enhancing their fatty acid metabolism, reducing inflammation and oxidative stress levels [20]. Caffeine can effectively curb weight increases in mice fed a high-fat diet [21], and its possible lipid-inhibiting mechanism may reside in regulating gut microbiota and its metabolites [22]. It was proved that either EGCG or CAF could reduce total lipids, triglycerides, and cholesterol in *Caenorhabditis elegans*, and their combined use was more effective than either alone [23]. Tian et al. [24] confirmed that both EGCG and ECG could reduce fat accumulation in *Caenorhabditis elegans*, thereby extending its lifespan. Thus, the WTAE likely exhibits potential lipid-lowering bioactivity and life-prolonging effects based on its composition.

### 3.2. Effects of the WTAE on the Lifespan of Flies Fed a High-Fat Diet

**Figure 2:**
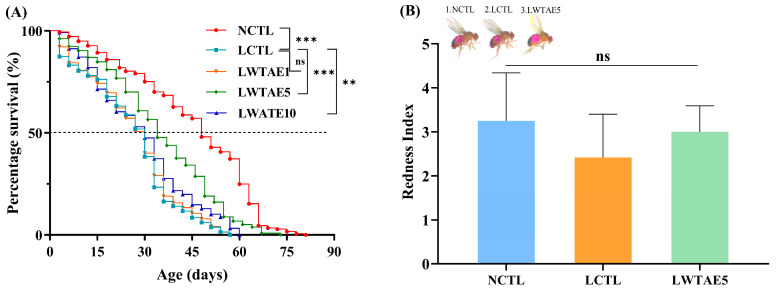

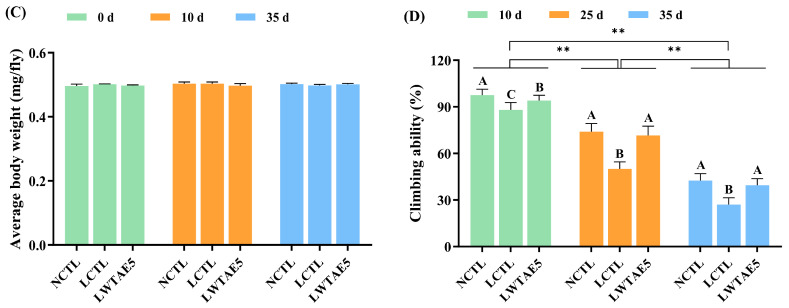
Effects of WTAE on lifespan (**A**), stomach redness index (**B**), average body weight (**C**), and climbing ability (**D**) of *Drosophila melanogaster*. ns (*p* > 0.05), ** (*p* < 0.01) and *** (*p* < 0.001) are assessed by log-rank Mantel–Cox tests for survival data. Data in (**B**–**D**) are expressed as mean ± SD. ns indicates no difference in the redness index among the flies in different feeding groups (**B**). Significant differences at *p* < 0.01 among groups at the same time are denoted by different capital letters, while differences in the same diet group at various times are expressed by ** (*p* < 0.01), all of which are assessed by one-way ANOVA followed by Tukey’s test.

The effects of the WTAE on the lifespan of male flies fed a lard diet were thoroughly investigated at three doses. The experimental results are presented in Figure 2A and Table 2. Compared to the NCTL group, the LCTL group experienced a substantial reduction in 50% survival time, mean lifespan, and maximum lifespan, with declines of 45.7%, 43.0%, and 25.3%, respectively. The introduction of dietary fat hastens the aging process in fruit flies, consistent with previous findings by Chen et al. [13] and Wang et al. [16]. Amazingly, the detrimental effects of a high-fat diet on flies’ mortality could be partially reversed when supplemented with WTAE. Among three intervention groups, flies exhibited the most remarkable improvement in the LWTAE5 group, with the 50% survival days prolonged from 22.4 days to 33.9 days, the mean lifespan extended from 25.9 days to 35.2 days, and the maximum lifespan enhanced from 50.5 days to 61.4 days (all *p* < 0.001). Furthermore, these considerable increases in lifespan were also observed in the LWTAE10 group (all *p* < 0.01), while no notable disparity was seen between the LWTAE1 and LCTL groups (*p* > 0.05). Due to these results, fruit files fed on WTAE5 were selected for subsequent experiments.

### 3.3. Effects of the WTAE on Feeding and the Average Body Weight of Flies

It has been verified that dietary restriction (DR) is one of the most effective measures for extending the lifespan of fruit flies [25]. To ascertain that the longevity effect observed in flies was not attributed to DR, we conducted an abdominal redness experiment on flies fed different diets. In this experiment, acid red was added to the diets to monitor changes in the feed intake of the flies. Additionally, alterations in the body weight of fruit flies were another indicator of feed intake. As depicted in Figure 2B,C, no significant disparity in food intake was observed among the NCTL, LCTL, and LWATE5 groups, as indicated by measurements of gustatory and body weight assays (*p* > 0.05). Therefore, we excluded the impact of DR on *Drosophila*.

### 3.4. Effects of the WTAE on the Climbing Ability of Flies

Aging involves reduced physical activity and age-related functional, metabolic, and structural decline in *Drosophila* skeletal muscle [26]. Climbing ability naturally declines during the normal aging process in fruit flies [27]. As shown in Figure 2D, a significant decline was presented in climbing ability with age across all three groups (*p* < 0.01). Additionally, the inclusion of 10% lard in the diet resulted in a notable decline in the climbing ability of flies (*p* < 0.01), a trend consistent with a previous study that revealed that exposure to a high-fat diet exacerbates this deterioration [28]. Interestingly, the decline in climbing ability caused by the lard diet was partially or completely reversible when supplemented with WTAE (*p* < 0.01). This observation aligned with previous results on incorporating bioactive peptides from crimson snapper and purple sweet potato anthocyanin into a high-fat diet [13,16].

### 3.5. Effects of the WTAE on Antioxidant Enzyme Activity in Flies

**Figure 3:**
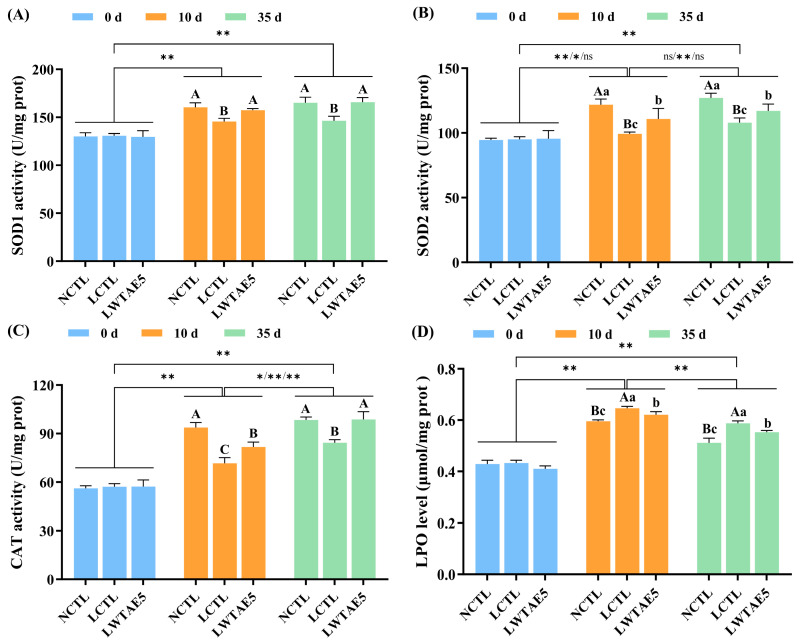
SOD1 (**A**), SOD2 (**B**), and CAT (**C**) activities and LPO levels (**D**) in *Drosophila melanogaster* reared on a non-lard control diet (NCTL), a 10% lard control diet (LCTL), or a 10% lard diet with 5 mg/mL white tea aqueous extract (LWTAE5) at 0, 10, and 35 days. Data are expressed as mean ± SD. Significant differences at *p* < 0.05 or *p* < 0.01 among groups at the same time are denoted by different lowercase or capital letters, while differences in the same diet group at different times are expressed by ns, *, or ** (*p* > 0.05, *p* < 0.05, and *p* < 0.01, respectively). All of these are assessed by one-way ANOVA followed by Tukey’s test.

One of the detrimental effects of aging arises from the increased generation of free radicals, which leads to oxidative damage in cellular structures and functions [29]. Organisms possess an antioxidant defense mechanism to counteract oxidative stress, primarily facilitated by antioxidant enzymes including SOD1, SOD2, GSH-Px, and CAT, which help maintain the balance of free radicals via scavenging excess radicals [30]. The present study assessed the effects of WTAE supplementation on the activities of SOD1, SOD2, and CAT in *Drosophila* reared on a lard diet. As depicted in Figure 3A–C, the activities of SOD1, SOD2, and CAT tended to increase initially and then stabilize with age in *Drosophila*. However, the levels of antioxidant enzymes have been reported to decrease with age [31], while Ma et al. [32] have observed that the activities of antioxidant enzymes (SOD1 and CAT) in *Drosophila* exhibited an initial increase and then decreased with age. This discrepancy can be attributed to the variation in time points for determining enzyme activities, or perhaps there are specific defense mechanisms within *Drosophila* when it is subjected to stress, resulting in an initial increase and then a decrease in the activities of antioxidant enzymes.

Intriguingly, a significant decline in the activities of SOD1, SOD2, and CAT was observed in flies fed a high-fat diet in comparison to those on a non-fat diet (all *p* < 0.01), with a reduction of 9.22%, 18.45%, and 23.57% on the 10th day, respectively, and a decrease of 11.29%, 15.04%, and 14.33% on the 35th day, respectively. However, these enzyme activities were partially or completely restored following WTAE intervention in a lard diet (*p* < 0.05 or *p* < 0.01), with an increase of 8.07%, 11.55%, and 14.09% on day 10, respectively, and an enhancement of 13.26%, 8.41%, and 17.13% on day 35, respectively. These results align with the discoveries made by Chen et al. [13], who also observed a decrease in antioxidant enzyme activities due to a high-fat diet. Nonetheless, they reported that supplementation with bioactive peptides originating from crimson snapper in a high-fat diet partially restored enzyme activities on the 21st day. Indeed, dietary supplementation rich in natural antioxidants can enhance the activities of antioxidant enzymes, thereby regulating oxidative stress [33].

### 3.6. Effects of the WTAE on LPO Levels in Flies

LPO, a critical marker of oxidative stress, serves as a widely employed indicator for assessing the extent of oxidative damage [34]. It is well-known that adding dietary fat into the diet can lead to the generation of lipid hydrogen peroxide, ultimately resulting in a shorter lifespan in *Drosophila* [16]. In this experiment, the evaluation of LPO production in fruit flies was performed using a specialized kit to explore the potential protective effect of WTAE against oxidative damage induced by lard in flies. As depicted in Figure 3D, the LPO levels in *Drosophila* initially rose and then declined with aging, and this trend was determined to be statistically significant (*p* < 0.01). Comparatively, the LCTL group displayed a considerable rise in LPO production of 8.39% and 11.69% on the 10th and 35th day, respectively, in comparison to the NCTL group. Remarkably, the addition of WTAE to a lard diet led to a significant reduction in LPO levels by 3.58% and 5.85% on day 10 and day 35, respectively (both *p* < 0.05). The current study unequivocally demonstrated that WTAE effectively inhibited lipid oxidation, partially mitigated LPO formation, and subsequently enhanced the survival rate of *Drosophila* given a high-fat diet. These findings indicated the antioxidant activity of WTAE in vivo in *Drosophila*.

Furthermore, our results disclosed a negative association between the levels of LPO in *Drosophila* and the levels of antioxidant enzymes at different ages. Enhanced activities of antioxidant enzymes correspond to reduced LPO levels, indicating that higher antioxidant enzyme levels are related to lower oxidative stress in cells [35]. It is noteworthy that the lifespan of *Drosophila* is closely linked to oxidative stress [36], which could explain the observed longer lifespan in *Drosophila* given a lard diet following WTAE treatment.

### 3.7. Transcriptome Profiles of Fruit Flies on Different Diets

**Figure 4:**
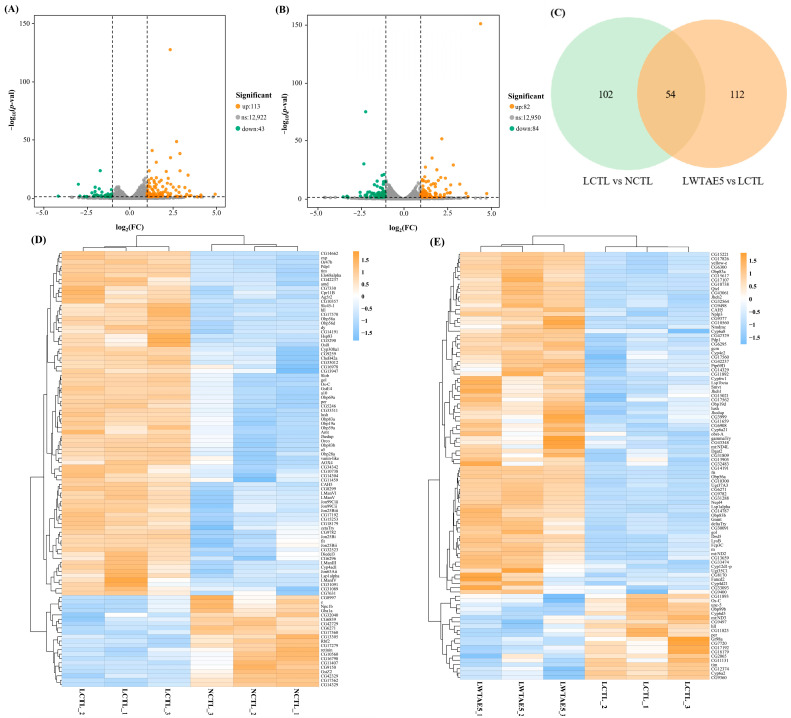
Treatment with WTAE regulates various gene expressions in aged flies induced by a high-fat diet. Volcano plots (**A**,**B**) represent the number of differentially expressed genes in flies between the LCTL and NCTL groups and between the LWTAE5 and LCTL groups, respectively. Venn diagram (**C**) presents the number of common and unique differentially expressed genes in flies between the LCTL vs. NCTL and LWTAE5 vs. LCTL groups. Heatmaps (**D**,**E**) show the clustering analysis of the top 100 differentially expressed genes in flies between the LCTL and NCTL groups, and between the LWTAE5 and LCTL groups, respectively.

To comprehensively explore the anti-aging mechanism of WTAE in high-fat diet-fed fruit flies, we conducted RNA sequencing (RNA-seq) on flies subjected to NCTL, LCTL, and LWTAE5 diets. A tota of 379,459,150 raw reads were obtained, which were subsequently filtered to yield 356,223,480 clean reads (93.88%). Approximately 92.41% to 94.80% of the clean libraries were successfully mapped to the *Drosophila melanogaster* reference genome (Table S2). The sequencing database provided over 13,000 gene expression profiles, with 80% or more of genes expressed in all three groups (Figure S1). Notably, the transcript levels of approximately 156 genes were significantly modified under high-fat diet conditions (113 genes were upregulated and 43 genes were downregulated in the LCTL group compared to the NCTL group) (Figure 4A). Simultaneously, the expression of 166 key genes saw significant changes following prolonged WTAE intake (82 genes were upregulated and 84 genes were downregulated in the LWTAE5 group compared to the LCTL group) (Figure 4B). Among these DEGs, 54 common coding genes were observed in data comparisons between the LCTL and NCTL groups, as well as between the LWTAE5 and LCTL groups. Furthermore, 102 and 112 DEGs were identified in the comparisons between the LCTL and NCTL groups and between the LWTAE5 and LCTL groups, respectively (Figure 4C).

### 3.8. Differentially Expressed Genes in Response to Different Diets

#### 3.8.1. Attenuated Expression of Genes Related to Fat Accumulation in Flies

*AOX4* is an isoenzyme encoding aldehyde oxidase, and *AOX4* knockout in mice results in resistance against diet-induced obesity and hepatic steatosis, which is due to the transformation of white adipocytes to brown adipocytes, thereby increasing energy expenditure [37]. Lipidomics analysis confirms that RNAi-mediated knockdown of *hll* expression significantly reduced fat storage [38]. According to our findings, *AOX4* and *hll*, involved in the peroxisome pathway and fatty acid biosynthesis and degradation pathway, respectively, were significantly upregulated in flies given a high-fat diet, while WTAE intervention significantly reversed the expression level of these two genes (Figure 4D,E; Table S3), suggesting that WTAE may inhibit fat accumulation in flies by reducing *AOX4* and *hll* expression.

#### 3.8.2. Suppressed Expression of Stress Response Genes in Flies

Heat shock proteins, intracellularly synthesized proteins of high conservation and ubiquitous presence, are induced in reaction to physiological, pathological, and environmental stresses such as heat or cold and dietary stimuli [39]. As molecular chaperones, they participate in the degradation and clearance of damaged proteins via the ubiquitin-proteasome or the autophagy-lysosome system [40]. Following a high-fat diet, heat shock protein genes involved in protein processing in the endoplasmic reticulum (ER) signaling pathway, such as *Hsp70Aa*, *Hsp70Ab*, and *Hsp83*, were significantly upregulated (Figure 4D; Table S3), indicating the induction of ER stress by a high-fat diet and the upregulation of heat shock proteins to counteract lipid stress. Our result is in accordance with a prior report, in which a high-fat diet induces increased expression of *Hsp83* in *Drosophila melanogaster* [41]. However, overexpression of heat shock proteins may surpass the cellular protective capacity against ER stress, ultimately leading to the activation of apoptosis mechanisms [42], and excessive expression of heat shock proteins has been confirmed to be associated with shortened lifespan related to an obesity-inducing diet [43]. Notably, an administration of WTAE to the LCTL group brought about a considerable reduction in the level of *Hsp83* transcripts (Figure 4E; Table S3). Similar results have been reported, where flies in a high-fat diet group, supplemented with Gomisin N, were capable of downregulating the heat shock protein *Hsp90* family member (*dGRP94*), which was thought to help extend the flies’ lifespans [44]. Hence, the potential of WTAE to mitigate high-fat diet-induced ER stress may contribute to the extension of fruit fly lifespan. Glutathione S-transferases (GSTs), as multifaceted enzymes, play pivotal roles in exogenous biological detoxification and anti-oxidative stress [45]. The findings of our study revealed that *GstE4*, implicated in the glutathione metabolism signaling pathway, was markedly upregulated in flies offered a high-fat diet and significantly downregulated upon WTAE intervention (Figure 4D; Table S3), mirroring the downregulation of *GstD2* and *GstE1* observed following anthocyanin intervention [46], suggesting the induction of oxidative stress by a high-fat diet and the potential amelioration of oxidative stress with WTAE supplementation.

#### 3.8.3. Regulation of Circadian Genes in Flies

In *Drosophila*, the circadian transcriptional activators Clock and Cycle drive the rhythmic expression of numerous target genes, among which are *period (per)* and *timeless (tim)* that encode repressors of Clock and Cycle activity [47]. Notably, previous research has proposed that a high-fat diet can induce alterations in the expression level of circadian clock genes, including *per*, *tim*, and *clock* (*clk*) in *Drosophila*, with *per* and *tim* showing increased expression at specific time points compared to the control group [48]. Moreover, these clock genes are associated with metabolic physiology, and their disruption causes metabolic phenotypes such as obesity and insulin resistance in animals [49,50]. Our findings implicated that *per* and *tim* were significantly upregulated following lard intervention, whereas their expression was markedly downregulated after WTAE supplementation (Figure 4D,E; Table S3). A recent study demonstrates that loss of specific circadian clock components (*per* and *tim*) in male *Drosophila* markedly increases lifespan via regulating intestinal mitochondrial uncoupling [47]. Thus, it is plausible to suggest that the downregulation of *per* and *tim* in flies fed WTAE may contribute to their extended lifespans compared to those given a high-fat diet.

#### 3.8.4. Enhanced Expression of Energy Metabolism Genes in Flies

The respiratory chain complex I constitutes the largest multi-protein enzyme complex in the mitochondrial electron transport chain [51]. Among its components, *mt:ND2*, *mt:ND3*, and *mt:ND4L* represent three of the seven genes encoding subunits of mitochondrial complex I (NADH-ubiquinone oxidoreductase), which is pivotal for catalyzing NADH dehydrogenation and facilitating electron transfer to ubiquinone for ATP production, which is essential for cellular respiration and oxidative phosphorylation [52]. These genes, integral to oxidative phosphorylation pathways, showed upregulation in fruit flies subjected to a high-fat diet supplemented with WTAE (Figure 4E; Table S3). Similarly, an increase in *mt:ND4L* expression was noted in male fruit flies on a high-fat diet supplemented with EGCG [53]. Knockdown of endogenous NADH dehydrogenase subunit 2 (ND2) significantly reduced intracellular ATP levels, elevated ROS levels, and diminished mitochondrial complex I enzyme activity [54]. Therefore, it was postulated that WTAE intervention may enhance the expression of genes encoding NADH dehydrogenase subunits in high-fat diet-fed flies, potentially ameliorating mitochondrial complex I enzyme activity, elevating ATP levels, and mitigating ROS levels, consequently fostering energy metabolism and hindering fat accumulation.

#### 3.8.5. Enhanced Expression of Neuroregulation Genes and Other Genes

Notably, three genes, *gammaTry*, *deltaTry*, and *gcm*, which are involved in nervous system regulation, were significantly upregulated in flies fed a lard diet following WTAE intervention (Figure 4E; Table S3). The first two genes participate in the neuroactive ligand–receptor interaction pathway that is accountable for the function of certain neurons and is predominantly engaged in the assembly of receptors and ligands on the plasma membrane [55]. This pathway is closely linked to memory function and sleep enhancement [56]. Experimental evidence indicated that an obesity-inducing diet negatively impacted healthy sleep patterns [43], potentially leading to decreased lifespan in fruit flies due to disrupted sleep homeostasis [57]. *gcm* is implicated in the differentiation of *Drosophila* glial cells (astrocytes and oligodendrocytes), which can differentiate glial progenitors into both neurons and glial cells [58]. Studies have shown its ability to alleviate inflammation triggered by Toll pathway activation in *Drosophila* hemocytes [59]. *Drosophila* glial cells function as a metabolic sensor, mobilizing peripheral lipid stores to maintain the brain’s metabolic homeostasis [60]. Hence, WTAE supplementation activates the neuroactive ligand–receptor interaction pathway and promotes glial cell differentiation, thus extending the lifespan of high-fat diet-fed fruit flies.

NAD-dependent methylenetetrahydrofolate dehydrogenase-methenyltetrahydrofolate cyclohydrolase (NMDMC) is a bifunctional enzyme crucial for folate-dependent metabolism and highly expressed in cells with rapid proliferation. Overexpression of this enzyme in the fat body of fruit flies was adequate to extend lifespan, reduce mitochondrial ROS levels, and confer resistance to oxidative stress [61]. We observed that Nmdmc was upregulated in high-fat diet-fed flies supplemented with WTAE (Figure 4E; Table S3), underscoring the significance of WTAE in extending lifespan in these flies, consistent with phenotypic observations. Additionally, *Jheh1* and *Jheh2*, involved in insect hormone biosynthesis signaling pathways, were upregulated following WTAE intervention (Figure 4E; Table S3). Juvenile hormone epoxide hydrolase (JHEH) is involved in juvenile hormone degradation, insect development, and molting processes. The homolog of JHEH in *Penaeus vannamei*, *PvJHEH1*, has been implicated in the immune response of *Penaeus vannamei*, with its knockout reducing the survival rate of Penaeus vannamei infected with *Vibrio parahaemolyticus* [62]. This may imply a potential function for *Jheh1* in the immune response of fruit flies.

### 3.9. Enrichment Analysis for Differentially Expressed Genes

**Figure 5:**
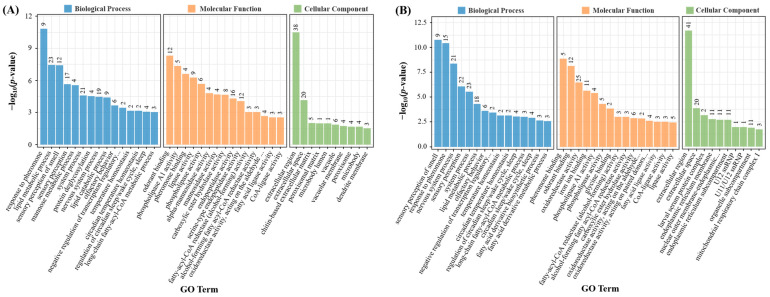

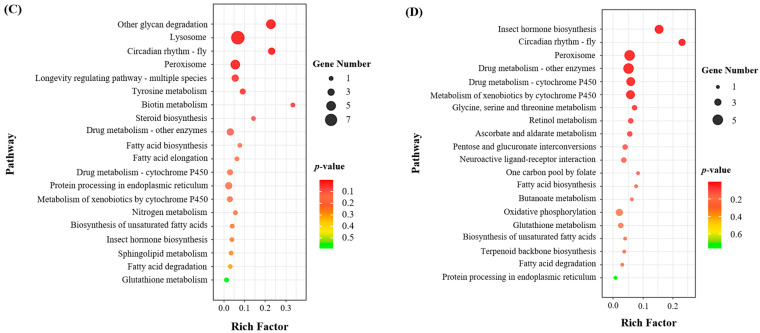
Bioinformatic enrichment analysis for differentially expressed genes. GO (**A**) and KEGG pathway (**C**) analysis of differentially expressed genes between flies fed with or without a lard diet. GO term (**B**) and KEGG pathway (**D**) analysis of differentially expressed genes between the high-fat diet flies fed with or without 5 mg/mL WTAE.

GO enrichment analysis highlighted that genes influenced by the high-fat diet were predominantly associated with diverse biological processes, including the response to pheromones, lipid metabolism, sensory perception of smell, sensory perception, mannose metabolism, system processes, protein deglycosylation, nervous system processes, lipid catabolism, long-chain fatty-acyl-CoA metabolism, etc. Additionally, these genes presented various molecular functions, such as phospholipase A1 activity, lipase activity, phospholipase activity, alpha-mannosidase activity, mannosidase activity, carboxylic ester hydrolase activity, etc. (Figure 5A). Upon intervention with WTAE, significant regulatory effects were observed in biological processes such as lipid metabolism, long-chain fatty-acyl-CoA metabolism, nervous system processes, and molecular functions including oxidoreductase activity (acting on the aldehyde or oxo group of donors), phospholipase A1 activity, fatty-acyl-CoA reductase (alcohol-forming) activity, fatty acid ligase activity, CoA-ligase activity, and lipase activity (Figure 5B).

The top 20 enriched KEGG pathways among DEGs in the LCTL versus NCTL group and the LWTAE5 versus LCTL group were depicted in Figure 5C,D. Pathways such as other glycan degradation, lysosome, circadian rhythm-fly, peroxisome, longevity regulating pathway-multiple species, tyrosine metabolism, and biotin metabolism were impacted by the lard diet. In contrast, the lard diet supplemented with WTAE influenced pathways related to insect hormone biosynthesis, circadian rhythm-fly, peroxisome, glycine, serine, and threonine metabolism, neuroactive ligand–receptor interactions, one carbon pool by folate, oxidative phosphorylation, and protein processing in the endoplasmic reticulum.

### 3.10. Validation of DEGs by qRT-PCR

**Figure 6:**
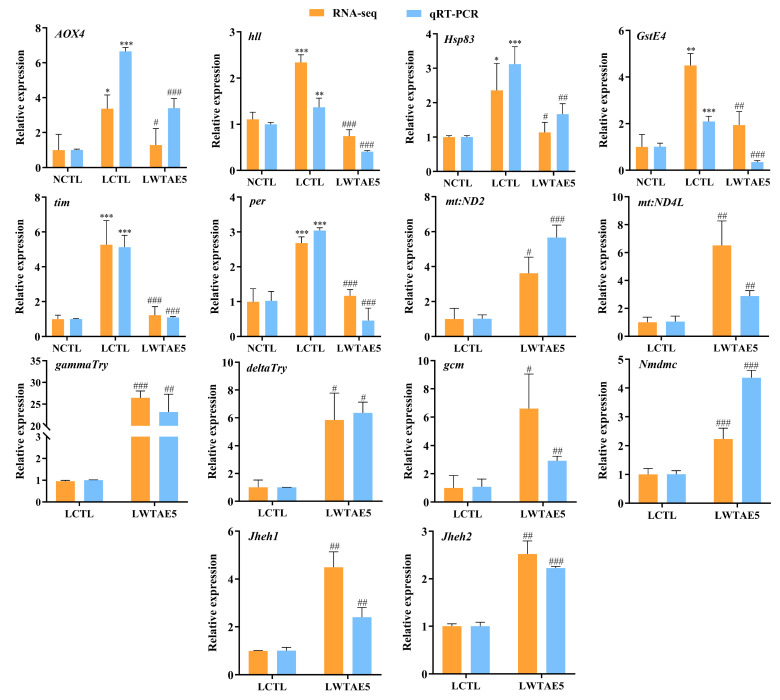
Validation of differentially expressed genes with qRT-PCR (mean ± SD). * *p* < 0.05, ** *p* < 0.01, and *** *p* < 0.001 vs. NCTL; ^#^ *p* < 0.05, ^##^ *p* < 0.01, and ^###^ *p* < 0.01 vs. LCTL.

To confirm the reliability of the RNA-seq results, we meticulously chose 14 candidate genes associated with the resistance to aging induced by a lard diet for qRT-PCR analysis. The findings revealed a congruence between the qRT-PCR assessment and the RNA-seq results (Figure 6), providing additional evidence for the dependability of our integrated omics and targeted screening strategy.

## 4. Conclusions

Our comprehensive experimental analysis demonstrated that WTAE remarkably extended the mean and maximum lifespan of *Drosophila* given a high-fat diet. Simultaneously, WTAE inclusion substantially improved *Drosophila*’s locomotor ability and elevated their antioxidant activity. Furthermore, WTAE may decelerate the aging process primarily by activating pathways such as oxidative phosphorylation, neuroactive ligand–receptor interaction, and the one-carbon pool by folate, while also inhibiting pathways including circadian rhythm-fly, protein processing in the endoplasmic reticulum, peroxisome, and fatty acid biosynthesis. Consequently, these results unveiled WTAE as an effective candidate for combating aging-related effects in the context of high-fat diets.

## Figures and Tables

**Table 1:** Major chemical constituents in the white tea aqueous extract (WTAE).

Chemical Constituents	White Tea Aqueous Extract
Total polyphenols (%)	22.92 ± 0.40
Total flavonoids (%)	2.24 ± 0.01
Free amino acids (%)	7.30 ± 0.02
Soluble proteins (%)	17.50 ± 0.23
Soluble sugars (%)	9.38 ± 0.43

Data are expressed as mean ± SD.

**Table 2:** Effects of WTAE at different doses on the lifespan of *Drosophila melanogaster* fed a high-fat diet.

Group	50% Survival (days)	Mean Lifespan (days)	Maximum Lifespan (days)
NCTL	44.9 ± 5.9	45.4 ± 5.4	67.6 ± 4.6
LCTL	24.4 ± 0.9 ^###^	25.9 ± 1.0 ^###^	50.5 ± 2.9 ^###^
LWTAE1	25.0 ± 0.8	26.7 ± 0.8	51.8 ± 2.1
LWTAE5	33.9 ± 4.5 ***	35.2 ± 4.1 ***	61.4 ± 6.8 ***
LWTAE10	27.6 ± 2.3 **	29.1 ± 2.2 **	55.8 ± 3.1 **

Data are expressed as mean ± SD. ^###^ *p* < 0.001 compared with the NCTL group of the corresponding lifespan. ** *p* < 0.001 and *** *p* < 0.001 compared with the LCTL group of the corresponding lifespan (Student’s *t*-test).

## Data Availability

The original contributions presented in this study are included in the article/Supplementary Materials. Further inquiries can be directed to the corresponding author.

## Supplementary Materials

The following supporting information can be downloaded at: https://www.mdpi.com/article/10.3390/foods13244034/s1. Table S1: List of forward and reverse primers used in this study; Table S2: RNA sequencing mapped statistics; Table S3: Partial coding genes with differential expression in response to a lard diet or a lard diet supplemented with 5 mg/mL white tea aqueous extracts; Figure S1: Treatment with WTAE regulates various genes expression in aged flies induced by a high-fat diet.
