# Supplementary material for: White Tea Aqueous Extract: A Potential Anti-Aging Agent Against High-Fat Diet-Induced Senescence in Drosophila melanogaster
Source: Foods. 2024 Dec 13;13(24):4034. doi: 10.3390/foods13244034 (PMC11728359; doi:10.3390/foods13244034)
Supplement: Supplementary file 1 [file foods-13-04034-s001.zip › foods-3320118-supplementary material.pdf]

Table S.1 List of forward and reverse primers used in this study.

Genes	Forward (5'-3')	Reverse (5'-3')
AOX4	TCCACAATCCACGACCGAGTAG	ACACTAACAGCCAAACAGCAAGG
hll	CCTCGGGCACAGTGGGTATG	ACATGGCTCAGCGGAAGGTAG
Hsp83	AGGTGATCCGCAAGAACCTG	GAGGTGTGGAAGCGAAGGAA
GstE4	ATGTCTGCTCACCCTGAAGGC	CCGTGTGCTGTGGATTCTTCTTC
tim	AAGAGGCCAGCGATATGACG	CAGTTGCCTGTGTCTCTGGT
per	TACCCGCATCCTTCGCTTTT	TTGTTGTACGCGGATTGGGA
mt:ND2	AGATGCTGCCTCCAAGAACC	CACACACAGACAGACGGACA
mt:ND4L	TATTGACGCAGCCTGTGGAG	TTGTTGGCAGCTCTTGGTCT
gammaTry	TCACCTTCTCTGTGTCCTCCTTC	CGTTGATCTTGATGATGGCAATGTC
deltaTry	TCACCTTCTCTGTGTCCTCCTTC	CGTTGATCTTGATGATGGCAATGTC
gcm	GAGCAATGGAAGCCAAAGCC	TCCCTGTTGCTGATGTTGCT
Nmdmc	TGGACAAGGATGTGGATGGC	ATGCTCCAACAGTCGCTTGA
Jheh1	TGCCAGATGGAGGACTCACTAAG	GGTGGTAATCGAATTGGTCAGGTAG
Jheh2	ATCTGCCCAAATGGAGCGAA	GGCTTTTGGCCTTTCACCTG
Rp49	CTTCATCCGCCACCAGTC	GCACCAGGAACTTCTTGAATC

Table S.2 RNA sequencing mapped statistics. Clean Reads: Total number of sequences used for comparison; Total Mapped: the proportion of the reference genome compared; Uniquely Mapped: proportional to only one position; Mapped to Gene: proportion mapped to gene region.

Sample	Clean Reads	Total Mapped	Uniquely Mapped	Mapped to Gene
NCTL_1	35651002	33160430（93.01%）	32588706（98.28%）	32492918（99.71%）
NCTL_2	40217106	37566293（93.41%）	36881360（98.18%）	36770837（99.70%）
NCTL_3	37855306	35620397（94.10%）	34984839（98.22%）	34875058（99.69%）
LCTL_1	40425102	38323489（94.80%）	37602186（98.12%）	37490570（99.70%）
LCTL_2	38966678	36726749（94.25%）	36015146（98.06%）	35902648（99.69%）
LCTL_3	37598998	35433850（94.24%）	34655359（97.80%）	34551222（99.70%）
LWTAE5_1	39249908	36801825（93.76%）	36058618（97.98%）	35920477（99.62%）
LWTAE5_2	45424554	41977784（92.41%）	41175817（98.09%）	41040849（99.67%）
LWTAE5_3	40834826	38205995（93.56%）	37425999（97.96%）	37277268（99.60%）

Table S.3 Partial coding genes with differential expression in response to a lard diet or a lard diet supplemented with 5 mg/mL white tea aqueous extracts.

KEGG pathway	Gene ID	Gene Name	LCTL vs NCTL(*P*-value)	LWTAE5 vs LCTL(*P*-value)
Peroxisome	FBgn0038350	*AOX4*	↑0.00031	↓0.00374
Fatty acid biosynthesis	FBgn0286723	*hll*	↑9.89E-08	↓0.00020
Protein processing in endoplasmic reticulum	FBgn0013275	*Hsp70Aa*	↑0.037418264	–
	FBgn0013276	*Hsp70Ab*	↑0.047983332	–
	FBgn0001233	*Hsp83*	↑0.00243	↓0.00925
Glutathione metabolism	FBgn0063496	*GstE4*	↑1.01E-13	↓0.01169
Circadian rhythm - fly	FBgn0014396	*tim*	↑1.194E-128	↓5.18211E-76
	FBgn0003068	*per*	↑2.12043E-14	↓4.57818E-21
One carbon pool by folate	FBgn0010222	*Nmdmc*	–	↑8.05E-07
Oxidative phosphorylation	FBgn0013680	*mt:ND2*	–	↑0.00001
	FBgn0013681	*mt:ND3*	–	↑8.95E-13
	FBgn0013683	*mt:ND4L*	–	↑0.00001
Insect hormone biosynthesis	FBgn0010053	*Jheh1*	–	↑3.64E-19
	FBgn0034405	*Jheh2*	–	↑2.15E-15
Neuroactive ligand-receptor interaction	FBgn0010359	*gammaTry*	–	↑0.00003
	FBgn0010358	*deltaTry*	–	↑0.00001
–	FBgn0014179	*gcm*	–	↑0.00051
–	FBgn0010053	*Jheh1*	–	↑3.64063E-19
–	FBgn0034405	*Jheh2*	–	↑2.15159E-15

Fig. S.1. Treatment with WTAE regulates various genes expression in aged flies induced by a high-fat diet. Venn diagram (A) and venn diagram (B) showing the number of common and unique expressed genes in flies between the LCTL and NCTL groups, and between LWTAE5 and LCTL groups, respectively.
